# Prospective Analysis of Maternal Mortality in Konaseema, Andhra Pradesh: Risk Factors, Causes, and Healthcare Challenges

**DOI:** 10.7759/cureus.83259

**Published:** 2025-04-30

**Authors:** Varada A Hasamnis, Mounika Meesala, K. Vasudhabhargavi, Manjiri Babar, Rajulapudi Manideepika, Moutam Shailaja, Suresh Babu Sayana

**Affiliations:** 1 Obstetrics and Gynaecology, Konaseema Institute of Medical Sciences and Research Foundation, Amalapuram, IND; 2 Anatomy, Symbiosis Medical College for Women, Pune, IND; 3 Pharmacology, Government Medical College and General Hospital, Kothagudem, IND

**Keywords:** healthcare delays, konaseema, maternal health, maternal mortality, postpartum hemorrhage, puerperal sepsis

## Abstract

Background: Maternal mortality remains a significant public health concern in developing countries, where many maternal deaths are preventable with timely and effective care. Understanding the underlying risk factors, causes, and challenges within healthcare systems is essential for designing targeted interventions. This study aimed to investigate the determinants of maternal mortality in Konaseema, Andhra Pradesh, India, to identify key areas for improving maternal health outcomes.

Methods: This prospective study analyzed maternal mortality data from medical records at the District Medical and Health Office (DM & HO) in Mummidivaram over a 2.5-year period, from April 2022 to January 2025. The study focused on women who died due to pregnancy-related complications during pregnancy, childbirth (regardless of outcome: live birth, stillbirth, or abortion), or within 42 days following the end of pregnancy, consistent with the World Health Organization (WHO) definition of maternal mortality. A total of 62,750 deliveries and 62,713 live births were recorded during the study period, with 40 maternal deaths identified. Data on demographic characteristics, obstetric conditions, healthcare facility characteristics, causes of mortality, and types of delays were extracted and analyzed.

Results: In Konaseema, the maternal mortality ratio (MMR) stood at 63.78 per 100,000 live births. Most maternal deaths occurred among women aged 20 to 34 years, totaling 34 cases (85%), and among multigravida women, accounting for 23 cases (57.5%). The majority of deaths (32 cases, 80%) occurred in the postpartum period, and the leading complications included puerperal sepsis (seven cases, 17.5%), hypertension with pulmonary edema (five cases, 12.5%), and postpartum hemorrhage (five cases, 12.5%). Delays in seeking treatment, accessing healthcare facilities, and receiving timely care significantly contributed to mortality, with 18 cases (45%) experiencing a delay in the decision to seek assistance.

Conclusions: Despite improvements in healthcare infrastructure, maternal mortality in Konaseema remains high due to delays in care and referral from other healthcare centers. Strengthening referral systems, improving healthcare access, and enhancing healthcare worker training are essential to reducing maternal deaths. Addressing these delays through effective interventions could significantly improve maternal health outcomes in the region.

## Introduction

Maternal mortality remains a critical public health concern, particularly in low- and middle-income countries [1]. A death occurs approximately every two minutes as of 2023, with over 90% of case records found in these nations. As of 2020, there are generally 223 deaths per 100,000 live births. Infections, severe bleeding, high blood pressure during pregnancy, complications from pregnancy, and unsafe abortions are the most common causes [2]. The World Health Organization (WHO) defines maternal mortality as the death of a woman during pregnancy, childbirth, or within 42 days after the termination of pregnancy, regardless of the duration or location of the pregnancy [3]. In developing countries, a large proportion of maternal deaths are preventable with timely and effective healthcare interventions [4].

Despite global progress in reducing maternal mortality, it remains a significant challenge in India, where the national maternal mortality ratio (MMR) is 97 per 100,000 live births [5]. (MMR refers to the number of maternal deaths per 100,000 live births during a specified time period, whereas maternal mortality rate refers to the number of maternal deaths in a population of women of reproductive age (usually 15-49 years) per 100,000 women of reproductive age during a specified time period). In the state of Andhra Pradesh, the MMR remains relatively high (MMR <70) [6,7].

India has made significant progress in maternal health, featuring improved antenatal care (ANC) services, a rise in institutional deliveries, and better access to emergency obstetric care. Nonetheless, the country's MMR still exceeds the Sustainable Development Goal (SDG) objective of lowering it to under 70 deaths per 100,000 live births by 2030 [7]. As per the latest reports, Andhra Pradesh, where this study is based, has an MMR of 45 per 100,000 live births, which is better than the national average but still concerning [8]. Konaseema, a district in Andhra Pradesh, faces unique healthcare challenges due to its rural and remote geographical setting. The region's MMR, while lower (MMR ≅ 65) than the national average (MMR <70) [7], still reflects significant healthcare gaps, including delays in seeking care, inadequate referral systems, and limited access to emergency obstetric services. Identifying the root causes and risk factors of maternal mortality in this region is crucial for developing targeted interventions that enhance maternal health outcomes.

This study aims to analyze maternal mortality data from Konaseema, focusing on identifying the primary causes of death, healthcare challenges, and delays in receiving appropriate care (higher age at first birth, higher body mass index (BMI), anemia, urban population proportion, and higher scheduled caste (SC)/scheduled tribe (ST) population proportion). By exploring these factors, the study seeks to provide valuable insights to improve maternal health strategies in the region and reduce preventable deaths.

## Materials and methods

Study design

This study is a prospective cross-sectional analysis aimed at investigating maternal mortality in the Konaseema district of Andhra Pradesh, India. The data was collected from medical records available at the District Medical and Health Office (DM & HO) in Mummidivaram over 2.5 years, from April 2022 to January 2025. The study focused on maternal deaths that occurred at any gestational age, whether during pregnancy, childbirth (including live birth, stillbirth, or abortion), or within 42 days of the termination of pregnancy, consistent with the WHO definition. This included deaths occurring during hospitalisation or post-discharge.

Study population

The study population comprised women who died from complications related to pregnancy during the study period. This encompassed women who gave birth in hospitals, at home, or who were referred from other healthcare centers. The study excluded deaths due to accidental or incidental causes, such as violence or external trauma, as these do not fall under the WHO definition of maternal death.

Data collection

Comprehensive information was gathered from case audits located in the medical records section of the DM & HO office in Mummidivaram. This data encompassed demographic details (age, gravida, residence), obstetric factors (ANC, booking status, pregnancy complications, mode and place of delivery), characteristics of healthcare facilities (referral status, ICU admission, duration from admission to death), as well as causes and locations of mortality. Further information was collected regarding various types of delays in accessing healthcare [9], such as delays in seeking care, travelling to healthcare facilities, and obtaining proper treatment at those facilities.

Variables analyzed

The study analyzed several key variables: (i) demographic characteristics, including age, number of pregnancies (gravida), and residence; (ii) obstetric characteristics, including ANC, booking status, complications during pregnancy, mode of delivery, place of delivery, delivery complications, and timing of admission; (iii) healthcare facility characteristics, including referral status, ICU admission, time, and place of mortality; (iv) causes of maternal mortality - both primary and contributory causes of maternal death, such as sepsis, hemorrhage, hypertension, and embolism; and (v) types of delays - classified into four types: Delay 1 (delay in decision to seek care), Delay 2 (delay in traveling to healthcare facilities), Delay 3 (waiting for care due to insufficient resources or skills), and Delay 4 (no delay).

Data analysis

Data were entered into a Microsoft Excel spreadsheet (Microsoft Corp., Redmond, USA) and analysed using IBM SPSS Statistics software, version 24 (IBM Corp., Armonk, USA). Descriptive statistics, such as frequencies and percentages for categorical variables, illustrated the study population's characteristics. The MMR was determined by the number of maternal deaths per 100,000 live births during the study period.

Ethical considerations 

The study was initiated after obtaining approval from the Institutional Ethical Committee (IEC), Konaseema Institute of Medical Sciences and Research Foundation, Amalapuram (IEC/PR/2021: 01/1/01.03.2022). The research maintained the confidentiality of all data gathered from medical records. The study's purpose was communicated to the appropriate authorities, and approval was secured for data retrieval.

## Results

The study intended to identify the demographic characteristics, obstetric characteristics, healthcare facility features, causes of maternal mortality, and the delays contributing to maternal mortality in Konaseema, Andhra Pradesh. A total of 40 maternal mortality cases were reviewed.

Demographic characteristics

Table 1 summarizes the demographic characteristics of maternal mortality cases. The highest proportion of deaths (85%; n=34) occurred among women aged 20-34 years, while both the younger (<19 years) and older (>35 years) age groups accounted for 7.5% (n=3) each. In terms of parity, multigravida women (having 2-4 previous pregnancies) comprised 57.5% (n=23) of the cases, whereas primigravida women (first pregnancy) accounted for 42.5% (n=17). Particularly, all maternal deaths (100%; n=40) occurred among women residing in rural (terrain) areas.

**Table 1:** Demographic characteristics of maternal mortality

Variable	Frequency	Percentage
Age		
<19 years	3	7.50%
20-34 years	34	85%
>35 years	3	7.50%
Gravida		
Primigravida (1)	17	42.50%
Multigravida (2-4)	23	57.50%
Residence		
Terrain	40	100%

Obstetric characteristics

As shown in Table 2, 34 women (85%) received ANC, four (10%) did not receive any ANC, and two (5%) were unsure of their ANC status. Regarding booking status, 32 women (80%) were booked for delivery, while eight (20%) were not. The majority of the women, 34 (85%), were admitted during the antepartum period, and six (15%) were admitted postpartum. At the time of admission, 32 women (80%) were not in shock, whereas eight (20%) presented with shock. Complications during the index pregnancy were reported in 19 cases (47.5%), while 21 cases (52.5%) had no documented complications. In terms of gestational age at admission, two women (5.8%) were between 12 and 24 weeks, 11 (32.3%) were between 24 and 36 weeks, and 21 (61.7%) were admitted at term. Regarding mode of delivery, 23 women (57.5%) underwent cesarean section, three (7.5%) had vaginal deliveries, and eight (20%) had not delivered at the time of death. Deliveries occurred mainly at tertiary care hospitals and private nursing homes (11 cases or 27.5% each), followed by area hospitals (three cases or 7.5%) and community health centers (CHCs; one case or 2.5%). No home deliveries (0%) were recorded among the cases analyzed.

**Table 2:** Obstetric characteristics of maternal mortality CHC: Community health centers

Variable	Frequency	Percentage
Antenatal care		
Yes	34	85%
No	4	10%
Don’t know	2	5%
Booking status		
Booked	32	80%
Not booked	8	20%
State at admission		
Antepartum	34	85%
Postpartum	6	15%
Condition at admission		
Shock	8	20%
No shock	32	80%
Complications during index pregnancy		
Yes	19	47.50%
No	21	52.50%
Gestational age at admission		
12-24 weeks	2	5.80%
24-36 weeks	11	32.30%
Term	21	61.70%
Mode of delivery		
Vaginal delivery	3	7.50%
Cesarean section	23	57.50%
Not delivered	8	20%
Place of delivery		
Tertiary care hospital	11	27.50%
CHC	1	2.50%
Area hospital	3	7.50%
Private nursing home	11	27.50%
Home	0	0%

Healthcare facility characteristics

As shown in Table 3, most women, 30 (75%), were referred from other healthcare centers, while 10 (25%) were not. In 33 cases (82.5%), ICU admission was required, whereas seven women (17.5%) were not admitted to the ICU. Regarding the duration from admission to death, 29 women (72.5%) died within 24 hours, while 11 (27.5%) survived beyond 24 hours after hospital admission. The state at the time of death was predominantly postpartum in 32 cases (80%), with eight deaths (20%) occurring during the antepartum period. As for the place of death, 23 women (57.5%) died in tertiary care hospitals, 12 (30%) in non-tertiary care facilities, and five (12.5%) died in transit.

**Table 3:** Healthcare facility characteristics

Variable	Frequency	Percentage
Referral from other centers		
Referred	30	75%
Not referred	10	25%
ICU admission		
Yes	33	82.50%
No	7	17.50%
Admission to death duration		
<24 Hours	29	72.50%
>24 Hours	11	27.50%
State at the time of mortality		
Antepartum	8	20%
Postpartum	32	80%
Place of mortality		
Tertiary care	23	57.50%
Other than tertiary care	12	30%
In transit	5	12.50%

Causes of maternal mortality

As presented in Table 4, the leading cause of maternal mortality was puerperal sepsis, accounting for seven cases (17.5%). This was followed by cardiomyopathy, responsible for six cases (15%). Several other causes contributed to five deaths (12.5%) each, including hypertension with pulmonary edema, hemolysis, elevated liver enzymes, and low platelets (HELLP) syndrome, postpartum hemorrhage, and dengue with thrombocytopenia and disseminated intravascular coagulation (DIC). Pulmonary embolism was identified in three cases (7.5%) and amniotic fluid embolism in two cases (5%). Less common causes included sickle cell crisis, one case (2.5%), and acute respiratory distress syndrome (ARDS), one case (2.5%).

**Table 4:** Causes of maternal mortality HELLP: Hemolysis, elevated liver enzymes, low platelet count; DIC: Disseminated intravascular coagulation; ARDS: Acute respiratory distress syndrome

Cause of Mortality	Frequency	Percentage
Amniotic fluid embolism	2	5%
Pulmonary embolism	3	7.5%
Hypertension with pulmonary edema	5	12.5%
HELLP syndrome	5	12.5%
Postpartum hemorrhage	5	12.5%
Puerperal sepsis	7	17.5%
Dengue with thrombocytopenia and DIC	5	12.5%
Cardiomyopathy	6	15%
Sickle cell crisis	1	2.5%
ARDS	1	2.5%

Types of delays contributing to maternal mortality

Table 5 outlines the types of delays contributing to maternal mortality. The most common was Delay 1, involving a delay in seeking care, observed in 18 cases (45%). Delay 2, referring to delays in reaching healthcare facilities, accounted for 10 cases (25%). Regarding Delay 3, which includes delays in receiving appropriate care at healthcare facilities, two cases (5%) were due to a lack of supplies or equipment, three cases (7.5%) were related to inadequate skills of healthcare providers, and one case (2.5%) involved a delay in receiving treatment despite arrival at a facility. Notably, six women (15%) experienced no identifiable delays in accessing or receiving care, yet maternal death still occurred, underscoring the complexity and multifactorial nature of maternal health outcomes.

**Table 5:** Types of delays contributing to maternal mortality

Type of Delay	Frequency	Percentage
Delay 1: Delay in deciding to seek care	18	45%
Delay 2: Delay in reaching healthcare facilities	10	25%
Delay 3: Delay in receiving care		
Lack of supplies/equipment	2	5%
Inadequate skills of provider	3	7.50%
Delay in receiving treatment	1	2.50%
Delay 4: No delay	6	15%

This study highlights critical factors contributing to maternal mortality in Konaseema, including age, gravida status, complications during pregnancy, and delays in care-seeking behavior, reaching healthcare facilities, and receiving timely treatment.

## Discussion

This study investigated the determinants of maternal mortality, including risk factors, causes, and healthcare system challenges, in Konaseema, Andhra Pradesh, a region characterized by distinct healthcare dynamics. The MMR in Konaseema was recorded at 63.78 per 100,000 live births, lower than the national average of 97 per 100,000 live births, yet reflective of substantial scope for further improvement. This MMR is comparable to other studies in developing countries, where maternal deaths remain a significant public health issue, despite improvements in healthcare infrastructure [10].

Demographic and obstetric characteristics

The majority of maternal deaths occurred in women aged 20 to 34 years (34 cases; 85%) and in multigravida women (23 cases; 57.5%), aligning with findings from other studies that indicate higher maternal mortality among women in their reproductive years and those with multiple pregnancies. Although this age group is generally at lower risk for pregnancy complications compared to younger or older women, it remains highly susceptible to conditions such as hypertensive disorders, sepsis, and hemorrhage - all of which were identified as major causes of death in our study [11]. The high percentage of women who received ANC (34 cases; 85%) is a positive indicator of the accessibility of prenatal services in the region, yet the MMR remains concerning. This suggests that while ANC is essential, it is not sufficient on its own to prevent maternal deaths. The effectiveness of ANC in reducing maternal mortality depends on timely interventions, efficient referral systems, and access to emergency obstetric care [12].

Causes of maternal mortality

The primary causes of maternal mortality in Konaseema were puerperal sepsis (seven cases; 17.5%), hypertension with pulmonary edema (five cases; 12.5%), and postpartum hemorrhage (five cases; 12.5%). These findings are consistent with studies from other regions of India and sub-Saharan Africa, where obstetric hemorrhage, sepsis, and hypertensive disorders are among the leading causes of maternal death [13]. The fact that sepsis was the leading cause of mortality highlights the critical need for infection prevention, improved sanitation, timely administration of antibiotics, and enhanced management of postpartum care. Postpartum hemorrhage and hypertensive disorders - both of which are preventable or manageable with appropriate care - further underscore the importance of better monitoring during labor and the immediate postpartum period [14]. The fact that a significant proportion of deaths occurred in the postpartum period (32 cases; 80%) reinforces the need for close postpartum monitoring and timely intervention [15].

Delays and healthcare system challenges

The research indicated that delays in seeking medical attention, accessing healthcare facilities, and receiving timely treatment significantly contributed to maternal health issues and mortality. Notably, 45% of maternal deaths were attributed to delays in deciding to seek care (Delay 1), highlighting the need for targeted interventions to address this initial delay. Strategies such as community-based education programs, awareness campaigns on maternal danger signs, promotion of birth preparedness and complication readiness (BPCR) plans, and the engagement of community health workers to facilitate early recognition of pregnancy complications could significantly mitigate this delay. Involving male partners and family members in maternal health decision-making may further help reduce hesitancy in seeking timely care.

Additionally, 25% (10 cases) of deaths were caused by delays in reaching healthcare facilities (Delay 2). These findings align with studies from other developing countries, where such delays in care have been identified as major factors in preventable maternal deaths [16]. A significant portion of maternal deaths (30 cases; 75%) involved women who were referred from lower-level healthcare centers, underscoring gaps in the primary and secondary care systems. The lack of timely referral, particularly in rural and remote areas, often results in women arriving at tertiary care centers in a critically unstable condition, as evidenced by the 29 cases (72.5%) of deaths that occurred within 24 hours of admission. Addressing these delays through improved referral systems, better training of healthcare providers to recognize complications early, and enhanced transportation infrastructure could significantly reduce maternal mortality [17,18].

Strengthening the healthcare system

Despite the availability of emergency obstetric care in tertiary centers, the study identified several shortcomings, particularly in rural areas. These included inadequate resources, a lack of skilled personnel, and insufficient infrastructure, all contributing factors to maternal deaths. Strengthening the healthcare system at all levels - primary, secondary, and tertiary - is critical. Essential obstetric care services, including adherence to septic care bundles, implementation of massive transfusion protocols, timely surgical interventions, and effective management of hypertensive disorders, must be made more accessible and efficient. Additionally, enhancing the capacity of healthcare workers to recognize and manage obstetric emergencies can reduce the occurrence of preventable deaths [19,20]. Improving community awareness and promoting birth preparedness are also crucial, especially in rural areas where access to care is often delayed due to logistical challenges. Community mobilization, coupled with training healthcare workers to timely identify high-risk pregnancies and ensure appropriate referrals, can help lower maternal deaths [11,20].

This study is limited by its reliance on secondary data from medical records, which may have contained incomplete or inconsistently recorded information. The sample size was restricted to 40 maternal deaths over 2.5 years, limiting broader generalizability. The analysis did not include socio-economic, nutritional, or detailed clinical histories beyond the available records. Furthermore, the assessment of delays was based solely on documentation review without qualitative inputs from families or healthcare providers, which may not have fully captured the contextual factors influencing care-seeking behavior.

## Conclusions

This study emphasizes the critical factors contributing to maternal mortality in Konaseema, including delays in seeking care, reaching healthcare facilities, and receiving treatment. The primary causes of mortality were puerperal sepsis, hypertension, and hemorrhage, highlighting the need for improved medical interventions. Strengthening referral systems, enhancing access to emergency obstetric care, and addressing delays in care are essential strategies for reducing maternal mortality. Additionally, there is a need to improve ANC, booking status, and ensure timely intervention during high-risk pregnancies. Further research is necessary to identify targeted interventions and policies that can address these delays, improve healthcare access, and ultimately reduce maternal mortality in Konaseema and other rural regions in India.
